# Good rates of return-to-sport in athletes after revision anterior cruciate ligament reconstruction using autologous patellar tendon and lateral extra-articular tenodesis: a 2-year follow-up prospective study

**DOI:** 10.1007/s00590-023-03544-8

**Published:** 2023-04-11

**Authors:** Felipe Moreira Borim, Nayana Joshi Jubert, Maria Mercedes Revertè Vinaixa, Irene Portas-Torres, Joan Pijoan Bueno, Raquel Sevil Mayayo, José Vicente Andrés Peiró, Enric Castellet Feliu, Joan Minguell Monyart

**Affiliations:** 1 Surgery and Morphological Sciences, Universitat Autónoma de Barcelona (UAB), 08193 Bellaterra, Barcelona, Spain; 2 Orthopaedic Surgery Department; Knee Surgery Unit, Hospital Universitari Vall d’Hebron, Passeig Vall d’Hebron 119-129, 08035 Barcelona, Spain; 3 Reconstructive Surgery of Locomotor System Group - VHIR, Hospital Universitari Vall d’Hebron, Passeig de la Vall d’Hebron 119-129, 08035 Barcelona, Spain; 4 Bioengineering, Cell Therapy and Surgery in Congenital Malformations - VHIR, Hospital Universitari Vall d’Hebron, Passeig de la Vall d’Hebron 119-129, 08035 Barcelona, Spain; 5 Knee Surgery Unit, Clínica Corachan, Carrer de Buïgas, 19, 08017 Barcelona, Spain

**Keywords:** Anterior cruciate ligament, Autografts, Tenodesis, Revision, Knee

## Abstract

**Background:**

Most athletes who undergo revision of the anterior cruciate ligament reconstruction (ACLR) aim to return to their preinjury sport at a similar level of performance while minimizing the risk for reinjury. Additional lateral extra-articular tenodesis (LET) has recently been correlated with improved outcomes and low complication rate. Yet, there are few series evaluating return-to-sport (RTS) and clinical outcomes after revision ACLR using bone-patellar tendon-bone (BPTB) and LET in athletes.

**Methods:**

The study cohort consisted of 19 eligible athletes who had undergone their first revision ACLR using BPTB and LET (modified Lemaire) between January 2019 and 2020. Patients were prospectively followed and interviewed in a sports activity survey during a 2-year follow-up.

**Results:**

Despite all patients returning to sports after revision ACLR surgery, 52.6% resumed playing at their preinjury level. Furthermore, patient-reported functional outcomes improved significantly following revision surgery, as evidenced by improvements in IKDC [64.4 (± 12) to 87.8 (± 6)], Lysholm [71.27 (± 12) to 84.2 (± 9.7)], and SF-12 scales [Physical: 53.3 (± 3) 57 (± 1.2); Mental: 50.2 (± 3.3) to 52.7 (± 2.4)]. One case (5.3%) experienced persistent pain and underwent reoperation for a partial meniscectomy.

**Conclusion:**

After revision ACLR using autologous BPTB and LET, all active individuals are expected to RTS, similar to primary ACLR. The difference comes down to returning to the preinjury level, where the levels are lower depending on the sport and initial level of play. Good mid-term functional outcomes with a low complication rate can be expected in most cases.

**Study design:**

Case series; Level of evidence IV.

**Ethical Committee Approval Number:**

PR(ATR)79/2021 and HCB/2023/0173.

**Supplementary Information:**

The online version contains supplementary material available at 10.1007/s00590-023-03544-8.

## Introduction

Reconstruction of the anterior cruciate ligament (ACLR) is one of the most common surgical procedures, with a reported failure rate of 3–14% [1]. Patient outcomes are less favourable when failures occur, and they undergo revision procedures. These procedures have higher failure rates, complications, and poor functional outcomes [2, 3]. Most athletes who undergo revision ACLR aim to return to their preinjury sport at a similar level of performance while minimizing the risk of reinjury. However, for many, these goals are not always attained. Return-to-sport (RTS) rates after revision ACLR have a relatively high rate of RTS at any level (56–100%) but a relatively low rate of RTS at the preinjury level of play (13–69%) [4, 5].

Several factors, including recurrent instability, stiffness, and pain, may lead to less-than-expected results and prevent athletes from RTS [5]. Although the cause of rotational instability after revision ACLR is multifactorial, adding an extra-articular procedure is based on its ability to restrict rotational laxity [6]. Patient satisfaction, overall knee function, RTS, and functional scores appear to correlate more with the restoration of rotational stability than with translational stability, making it a critical short-term to mid-term goal [7, 8]. The limited body of evidence has shown that adding soft tissue procedures may lower the risk of graft re-rupture rates and improve overall outcomes [9]. Furthermore, compared to allografts, autografts have improved sports function, patient-reported outcome measures, and decreased graft re-rupture rate at a 2-year follow-up [3, 10].

In recent years, several studies [8, 11–22] have emphasized the crucial role of simultaneous revision ACLR using an autologous graft and LET in improving rotational stability and graft protection. Despite these findings, the scientific evidence remains highly heterogenous, with variations in patient selection, graft type, and surgical technique. To date, no study has specifically examined the RTS of athletes following revision ACL using concomitant autologous bone-patellar tendon-bone (BPTB) and LET (modified Lemaire), with a minimum follow-up of 2 years. Therefore, it is imperative to conduct a thorough analysis that primarily examines the assessment of RTS while also taking into account the clinical outcomes and potential complications that may arise in athletes who undergo this procedure. Our hypothesis suggests that by adopting this approach, we can anticipate positive outcomes in the short to mid-term, with good rates of RTS and minimal complications.

## Material and methods

### Patients recruitment and follow-up assessment

This study was approved by Hospital Universitari Vall d'Hebron and Hospital Clínic de Barcelona Ethics Committee, and patients signed informed consent before being included. All patients who underwent revision ACLR using autologous BPTB and modified Lemaire LET between January 2019 and January 2020 were screened for eligibility for this prospective study. Inclusion criteria were (1) age above 16 years and capable of giving consent for study participation; (2) patients with ACLR graft rupture diagnosed by clinical symptoms and physical exam, confirmed by magnetic resonance images (MRI); (3) Tegner Activity Scale Level ≥ 6 before primary ACL rupture and before primary ACLR. Exclusion criteria included (1) concomitant ligament injuries or coronal plane deformity; (2) incomplete follow-up and clinical data.

Patients' demographic, clinical and radiological data were collected preoperatively, postoperatively, and during the follow-up period until 24 months postoperatively. Patients were asked to complete a sports activity survey. The assessment included Tegner Activity Scale (TAS), International Knee Documentation Committee (IKDC) score, Lysholm Knee Score, and Short-Form Health Survey (SF-12) Physical and Mental. Concomitant lesions were recorded in radiological (radiographs, CT and MRI) and arthroscopic evaluation during the primary and revision surgery.

A total of 23 consecutive patients were initially screened; three were excluded for failing to meet the inclusion criteria; one case had an incomplete follow-up. At 24 months follow-up, 19 patients (men, n = 9; women, n = 10; mean age 27.7 years; SD 7.2) were available for follow-up. Two patients underwent a 2-stage revision and were also included.

Twenty-four-month outcome data for the cohort are available in the Appendix.

### Surgical technique

**Fig. 1:**
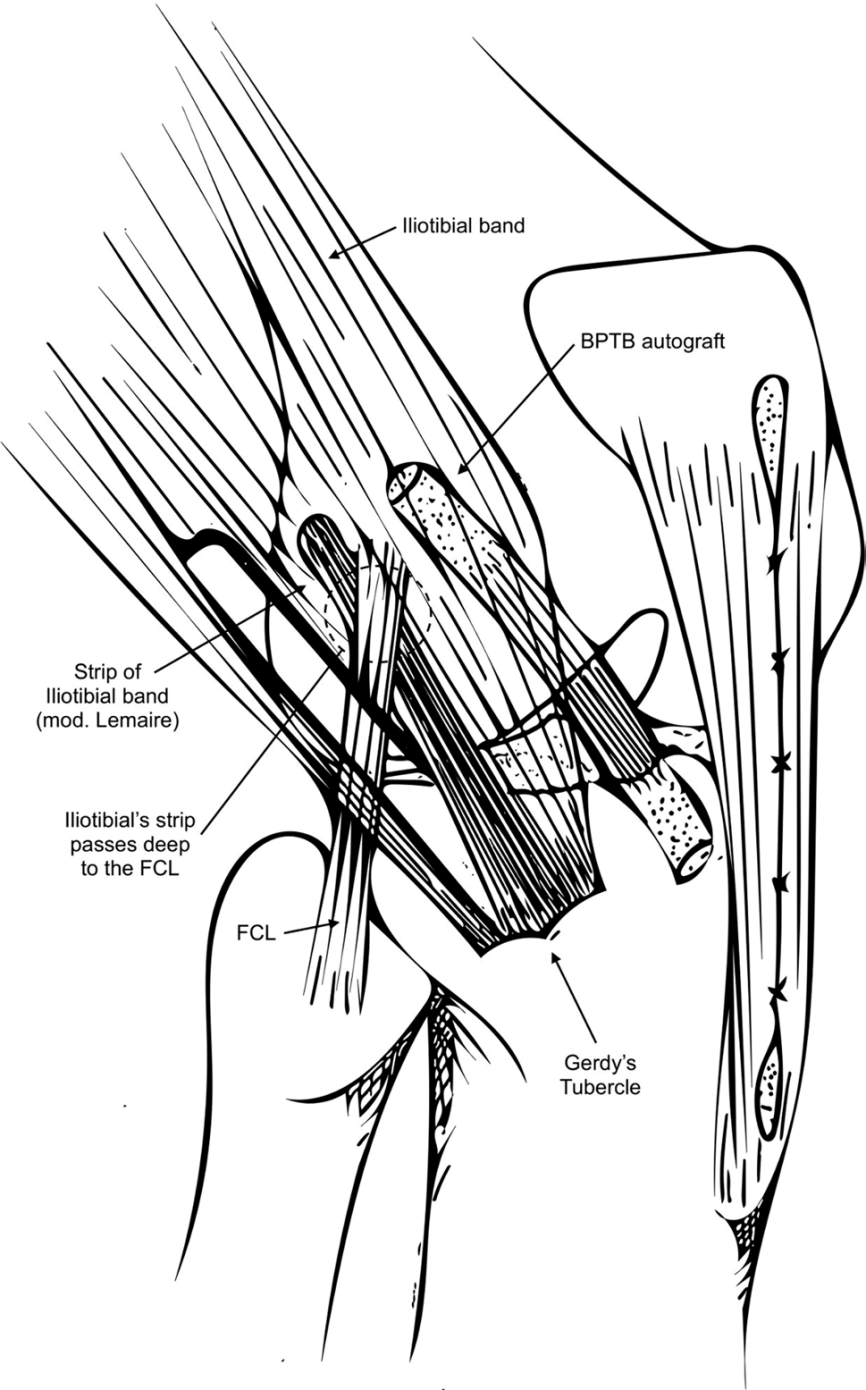
Schematic drawing of the lateral aspect of a right knee demonstrating a revision anterior cruciate ligament reconstruction (ACLR) using a bone–patellar tendon–bone autograft and a lateral extra-articular tenodesis procedure (modified Lemaire), with the iliotibial band autograft passed deep to the fibular collateral ligament (FCL), oriented with a 30° anterior angle in the axial plane and 30° proximal in the coronal plane

Combined spinal anaesthesia with regional nerve blockade was used. All patients were assessed under anaesthesia for ROM, Lachman, Pivot Shift, LCL, MCL, and pulse exams. A preliminary arthroscopic inspection was performed to help diagnose and treat associated meniscal and chondral injuries. Furthermore, the size of the intercondylar fossa is evaluated, and notchplasty is done if needed to avoid impingement. Progressive drilling of the tibial and femoral tunnels with cannulated drills of different sizes until completing the debridement of the previous graft site was done. BPTB autografts were prepared, usually 10 × 25 mm bone plugs; suspension systems were used for femoral fixation (TightRope® RT; Arthrex, Naples, FL), interferential screw (Biocomposite®; Arthrex, Naples, FL) and ligament staple were used for a hybrid fixation on the tibia (see Fig. 1). Lastly, a modified Lemaire LET was performed [23].

Patients were offered a two-stage surgery (1) if tunnel widening was so significant on both the tibia and femur that one-stage bone grafting is not feasible, usually enlarged over 14–16 mm; (2) malpositioned, which could result in tunnel overlapping; (3) arthrofibrosis or (4) local infection. The two-stage procedure involved an initial bone-allografting procedure and LET. Then an incorporation phase of 20–24 weeks, allowing the bone graft to heal before the subsequent second stage; a CT scan at 3–4 months was performed to confirm correct incorporation.

### Rehabilitation

For the first 4–6 weeks, walking with partial weight bearing was allowed using two crutches. Patients were encouraged to perform complete knee flexion and extension. Closed kinetic chain exercises and the use of a balance board to regain proprioception were performed for the first three months, and after that, open kinetic chain exercises were started. To authorize RTS, we considered physical and psychological aspects. In general, sports activities are allowed after a period of 9 months, but it is important to prioritize functional criteria when deciding to engage in them.

### Statistical analysis

Performed with Statistics STATA/IC 15.1 (StataCorp®). Categorical variables were described with their absolute values and percentages. Quantitative variables were presented by their measures of central tendency (mean and standard deviation). Preoperative and postoperative tests were compared using paired Mann–Whitney U tests. Differences with *p* values < 0.05 were considered statistically significant.

## Results

Table 1 provides a summary of the demographic data for the cohort of athletes who underwent revision ACLR using autologous BPTB and LET. Although all patients were able to return to some level of sports activity, the rate of return to preinjury level of sport was lower after the revision procedure. Out of the nineteen athletes, only ten (52.6%) were able to return to their preinjury level of sport, which is in contrast to the fifteen (79%) who were able to do so after primary reconstruction.

**Table 1:** Demographic data and concomitant lesions of the included patients (n = 19)

Patient data		
Sex, n (%) Female/male	10 (52.6%)	9 (47.4%)
Side, n (%) Right/left (%)	14 (73.7%)	5 (26.3%)
Age, y (SD)*	29.8 (± 7.5)	
Number of Stages, n (%) One-/two-stages	17 (89.5%)	2 (10.5%)

Concomitant lesions	Primary ACLR		Revision ACLR	
	n	%	n	%
Chondral lesion (medial, lateral and femoropatellar)	1	5.3	2	10.5
Meniscus lesion (medial, lateral)	6	31.6	9	47.4
Chondral or meniscal (any lesion)	7	36.9	11	57.9
No chondral nor meniscus lesion	12	63.1	8	42.1

Expressed as the n and (percentage). *Expressed in mean and (± SD)

*ACLR* anterior cruciate ligament reconstruction

**Fig. 2:**
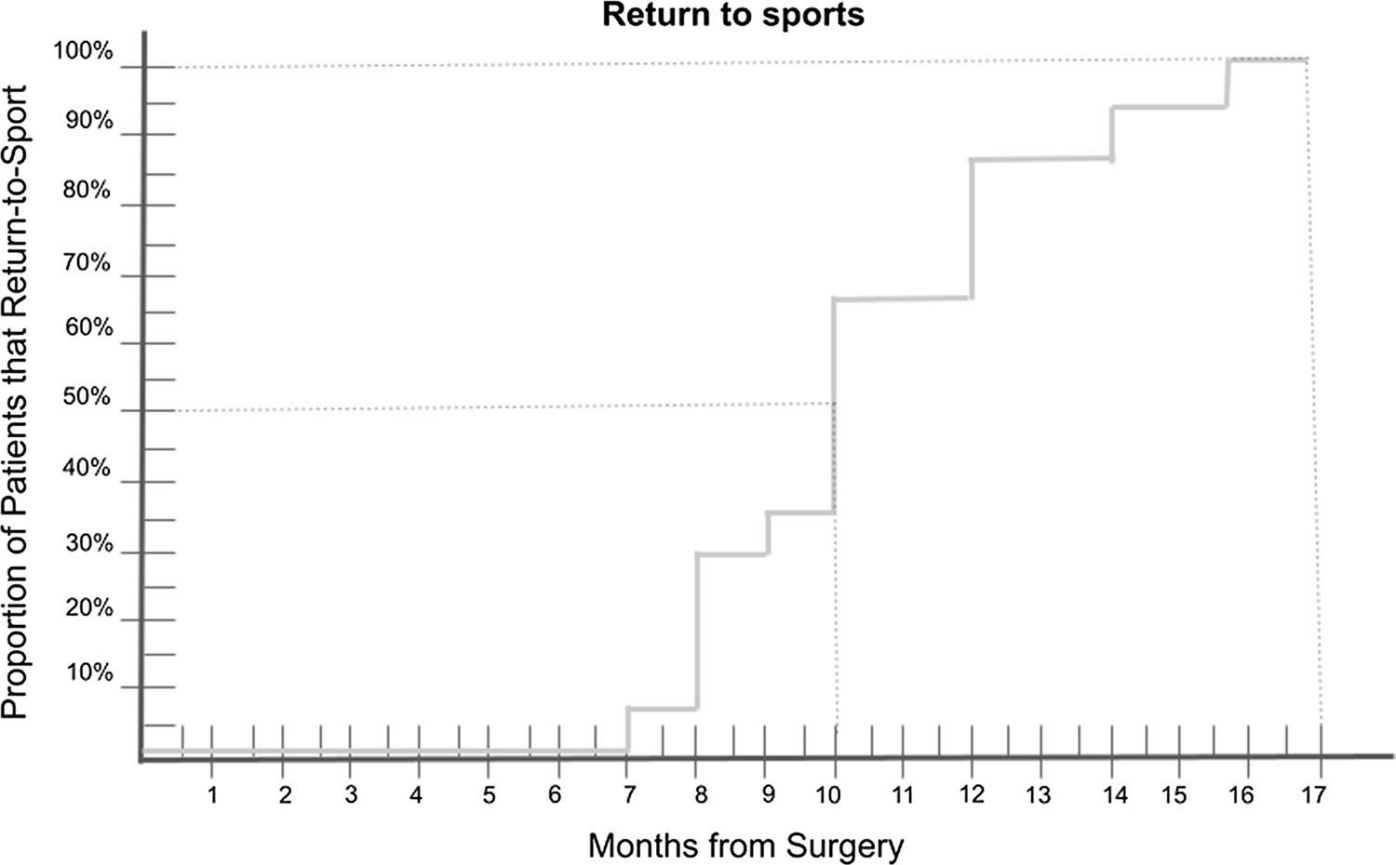
The proportion of patients that return-to-sport (RTS), measured in time in months

The majority of athletes (79%) participated in contact sports such as basketball and football, with only a few (15.8%) participating in noncontact sports like cycling and athletics, and one athlete (5.3%) involved in collision sports (rugby). Two professional athletes (10.5%) were included in the cohort, one of them did return to her preinjury level after the primary procedure, but not after the revision. The majority of patients (68.4%) were affiliated with a player's federation and engaged in regular sport. Age did not appear to have a significant influence on the return-to-sport (RTS) rate. Interestingly, the RTS rate for women was slightly higher (77.8%) than for men (55.6%). When selecting sports to participate in after revision ACLR, most patients opted for noncontact sports like padel (42.1%) and cycling (26.1%), with all of them also attending a gym for general fitness. Additionally, patients were asked to indicate the main reason why they did not return to their previous level of sport from among four categories, with knee-related reasons, such as pain or instability, only corresponding to 26.3% of the cases. Table 2 provides an overview of the RTS results. The mean time to return to sport was 10.3 months (as shown in Fig. 2).

**Table 2:** Return to sport (RTS) outcomes summary and comparison (n = 19)

Tegner Activity Score (TAS)*		
Before ACLR	7.4 (± 1)	(6–10)
After ACLR	7.1 (± 1)	(6–10)
Revision ACLR	6.4 (± 1)	(5–10)

List of sports	After primary ACLR		After revision ACLR	
Basketball	10	(52.6%)	6	(31.6%)
Football	6	(31.6%)	2	(10.5%)
Tenis/Padel	1	(5.3%)	7	(36.8%)
Athletics	1	(5.3%)	1	(5.3%)
Rugby	1	(5.3%)	1	(5.3%)
Cycling	–	–	2	(10.5%)

Level of return to sport			
	Same sport, same level	Same sport, lower level	Another sport, lower level
Total	10 (52.6%)	6 (31.6%)	3 (15.8%)
< 25 y	4 (57.1%)	2 (28.6%)	1 (14.3%)
≥ 25 y	6 (50%)	4 (33.3%)	2 (16.7%)
Reasons for changing or stopping the preinjury sport played			
Job-related^a^	Personal reasons^b^	Knee-related^c^	Medical reasons^d^
5 (26.3%)	6 (31.6%)	5 (26.3%)	2 (10.5%)

Values for outcomes are expressed expressed as n and (%). *Expressed in mean and (± SD)

*ACLR* anterior cruciate ligament reconstruction

^a^Job-related included work-related constraints

^b^Personal reasons included personal preferences or interests that might have changed

^c^Knee-related included those directly related to the limitations after the surgery, such as pain or instability

^d^Medical reasons included other conditions or indications that could prohibit the return

The functional improvement of patients was assessed using various scales. The IKDC score increased from 64.4 (± 12) to 87.8 (± 6), while the Lysholm score increased from 71.27 (± 12) to 84.2 (± 9.7). The SF-12 scale also showed significant improvement in both the Physical (from 53.3 ± 3 to 57 ± 1.2) and Mental (from 50.2 ± 3.3 to 52.7 ± 2.4) domains. However, the Tegner Activity Scale demonstrated a significant decrease in activity level from 7.2 (± 1) to 6.6 (± 1.1). An overview of these results can be found in Table 3.

**Table 3:** Patient-reported outcomes summary and comparison (n = 19)

Patient-reported outcome scales	Preoperative	24 month follow-up	*p* value
Lysholm^a^	64.4 (± 12)	87.8 (± 6)	0.0001
IKDC^b^	71.27 (± 12)	84.2 (± 9.7)	0.0005
SF-12 Physical^c^	53.3 (± 3)	57 (± 1.2)	0.0002
SF-12 Mental^c^	50.2 (± 3.3)	52.7 (± 2.4)	0.0025
TAS	7.2 (± 1)	6.6 (± 1.1)	0.0005

Expressed in mean and (± SD)

*TAS* Tegner Activity Scale

^a^Lysholm Knee Scoring System

^b^International Knee Documentation Committee subjective knee form

^c^Short Form (12) Health Survey

In addition, 57.9% of patients had concomitant lesions, which is a higher proportion than the 36.8% observed in the same group of patients who underwent primary ACLR. Table 1 provides a summary of the associated lesions. Notchplasty was performed in 6 cases (31.6%) due to intercondylar notch impingement.

Major complications accounted for one case (5.3%) of residual pain, where a partial meniscectomy was later performed. Minor complications accounted for 3 cases (15.8%), which included one case of hemarthrosis and two cases of material discomfort (see Table 1). Despite this, patient satisfaction and functional outcomes remained high, and no savage procedure was necessary. There were no cases of anteroposterior and rotational laxity detected (Lachman and Pivot-Shift tests) or reported by the patients. There was one case of contralateral primary ACL rupture.

## Discussion

The main finding of this cohort was that the addition of a LET to an autologous BTPB revision ACLR provides improved rates of RTS, patient-reported functional outcomes and minimal complications. Although all athletes did return to some level of play, only ten out of nineteen athletes (52.6%) could return to their preinjury level after a 2-year follow-up. Significant improvements were seen on Lysholm, IKDC and SF-12 Physical and Mental scales. When comparing the revision to the primary reconstruction, there was a notable increase in the prevalence of meniscus and osteochondral injuries, with the percentage rising from 36.9 to 57.9%. The rate of complications remained low, with only one case (5.3%) experiencing residual pain and reoperation for a partial menisectomy. To date, few studies have analysed the results of revision ACLR and LET [12–22], with differences in graft type and surgical technique. Furthermore, selecting a uniform group of patients presents a challenge. In an effort to achieve homogeneity, we carefully established our inclusion criteria.

This study presents some limitations. First, it is non-comparative, with the inherent biases of this type of study. Second, our cohort is small despite no consistent losses, and it has limited statistical power and, therefore, generalizability of the results. Third, the outcomes are not evaluated with objective radiological measurements, but with subjective tests and scales, with 24-month follow-up. However, our study provides information on using a single type of autograft combined with LET for revision ACLR in a very homogenous cohort.

There is a discrepancy between the rate of RTS at any level and the rate of return to sport to the preinjury level, which is more pronounced following revision ACLR [4, 5, 11]. Lefevre et al. (BTB/HS + extra-articular tenodesis with tensor fasciae latae tendon) compared RTS rates between patients who underwent primary ACLR and revision ACLR and found no significant difference in the rates of return to sport at any level between the two groups. However, the authors also found that athletes returned to their preinjury sport at a significantly higher rate after primary ACLR (64%) than after revision (49%) [12]. Similarly, in two recent systematic reviews, Grassi et al. reported that 53.4% (CI 37.8–68.7), and Glogovac et al. a range from 13 to 69%, of patients following revision ACLR had returned to the same sport at a preinjury level [4, 11]. Likewise, the rate of RTS at a preinjury level we saw in our cohort remained within that range at 52.3%. Table 4 summarises the results from the most important published studies on the subject to date.

**Table 4:** Summary from published studies on revisions ACLR using autologous grafts and LET: return to sport (RTS) and patient-reported functional outcomes

Authors	Year	Number of Patients	Autologous Graft, (%)	LET Technique	RTS Any Level, %	RTS Same Level, %	Time to Return, m	TAS	IKDC	Lysholm
Lefervre et al. [12]	2017	47 (n = 55)	HT (32.7%)/BPTB (52.7%)/CFL (14.6%)	Lemaire	87.3	49.1	7.4 (± 4.9)	–	–	–
								–	78.4 (16.6)	87.5 (12.9)
Alessio et al. [13]	2018	24	BPTB (79.9%)/HT (20.1%)	Coker–Arnold	100	91.7	9.2 (± 2.2)	9.5 (± 0.5)	69.5 (± 11.1)	58.1 (± 11.7)
								9.2 (± 1)	88.4 (± 8.9)	97.4 (± 3.2)
Redler et al. [14]	2018	118	HT	Coker–Arnold	100	41.5	–	3.6 (± 1.8)	70.3 (± 8.4)	67 (± 19.8)
								5.7 (± 1.9)	85.7 (± 12.3)	90 (± 7.2)
Legnani et al. [15]	2019	9	HT	Coker–Arnold	–	78	–	6 (± 2.8)	40.2 (± 4.2)	62.2 (± 3.4)
								6 (± 3.2)	85.6 (± 4.8)	87.9 (± 7)
Legnani et al. [16]	2019	12	HT	Coker–Arnold	–	58		6	35.7 (± 2.4)	60.8 (± 4.7)
								6	84.8 (± 6)	88.7 (± 6.1)
Alm et al. [17]	2020	59	HT/BPTB/QT	Lemaire	–	–	–	–	60 ± 23	–
								7 ± 1.3	95 ± 10.8	90 ± 10.7
Ventura et al. [18]	2021	12	HT	Coker–Arnold	–	–	–	6	35.7 (SD: 2.4)	60.8 (SD: 4.7)
								6	84.8 (SD: 6.0)	88.7 (SD: 6.1)
Eggeling et al. [19]	2021	23	HT (39.2%)/BPTB (30.4%)/QT (30.4%)	Lemaire	47.8	–	–	–	–	49.2 ± 28.1
								5.7 ± 1.3	77.5 ± 16.2	81.9 ± 14.2
Zanna et al. [20]	2022	17	BPTB	Coker–Arnold	94.1	58.8	–	–	71.4 (± 9.03)	58.3 (± 19.3)
								–	92 (± 6.9)	66.8 (± 27.7)
Rayes et al. [21]	2022	36	BPTB	Lemaire	86.1	61.1	–	–	54.4 (± 17.5)	64.8 (± 12.3)
								7.3	86.7 (± 10.6)	92.8 (± 10.5)
Keizer et al. [22]	2022	42	BPTB	Lemaire	52.3	30.9	–	–	–	–
								6	81.7 (± 13.4)	–
Case series (for comparison)	2023	19	BPTB	Lemaire	100	52.6	10.3	7.1 (± 1)	71.3 (± 12)	64.4 (± 12)
								6.4 (± 1)	84.2 (± 9.7)	87.8 (± 6)

Values for outcomes are expressed as mean and (SD). Studies that didn’t report on the use of autologous grafts and extra-articular augmentation (individually or by groups) were not included for comparison

*TAS* Tegner’s Activity Scale, *IKDC* International Knee Documentation Committee, *Lysholm* Lysholm Knee Score, *LET* lateral extra-articular tenodesis, *HT* hamstrings tendon, *BPTB* bone–patellar tendon–bone, *CFL* combined fasciae latae

There is undoubtedly significant heterogeneity in the results found in different studies [4, 11–22]. The reason for this is multifactorial but we believe it has a lot to do with the athletes' age, type of sport and initial level of play (elite, professional, or semi-professional). Our series was composed of professional and semi-professional athletes, and at a young age (median age 27.7 years). Younger age was associated with a higher rate of return to sport following revision ACLR in multiple studies [13–15]. When asked for reasons why they did not RTS at preinjury level only 26,3% attributed it “to the knee” and that it did not behave as well as before. Other reasons included personal reasons (31.6%), since the sport played did not represent the same importance in these patients' lives, professional reasons (26.3%) where work–life balance prohibited returning to the same training routines and 10.5% attributed to other medical conditions.

Many authors [6, 8, 9, 11–22] already advocate the critical role of adding an extra-articular procedure due to its ability to restrict rotational laxity [6]. Getgood et al. have recently found that adding LET to primary ACLR in young patients at high risk of failure results in a statistically significant reduction in graft rupture and persistent rotatory laxity two years after surgery. Some studies have looked into RTS after revision ACLR and LET [8, 11–22, 24], finding good functional outcomes, low rates of residual rotatory laxity, re-ruptures or complications. Louis et al. stated that combining ALL stabilization with revision ACLR improves functional outcomes by improving rotational stability without increasing the risk of early or late complications [24]. Alessio-Mazzola et al. [13] reported a RTS rate at preinjury level of 91.7% in professional soccer players and a mean time of return of 9.2 months. Similarly, we reported a 10.2 months mean time of return.

There are concerns about overtightening the lateral compartment during different LET techniques, which subsequently may lead to osteoarthritis [6, 9]. However, according to Declercq et al.'s comparison of modified Lemaire and Cocker-Arnold procedures, the choice of LET technique appears to have minimal impact on both clinical and radiographic outcomes [25]. In our series, we did not see any signs of overtightening or osteoarthritis.

There are inconsistent results in the literature regarding the impact of graft type on RTS outcomes [4, 11]. However, some reasons for favoring autografts are their improved patient-reported outcomes, RTS, and decreased graft re-rupture rate compared to allografts [3, 10]. Keizer et al. [26] retrospectively compared outcomes between patients with patellar tendon autografts and allografts, and after a follow-up of 2 years, the rate of RTS was 75% versus 43%, respectively. Shorter RTS times have also been reported [4, 11]. Moreover, Glogovac et al. [4] found that the studies that reported strictly patellar or hamstring tendon autografts demonstrated some of the highest rates of return to sport at preinjury levels (67–69%). In our case, unharvested BPTB has been our first choice for young and active patients, reserving contralateral grafts or allografts only in case of repeated revision, combined ligament reconstruction, or other particular extraordinary circumstances.

Revision ACLR procedures are known to be significantly more challenging and to present meniscal and cartilage injury in nearly 90% of patients [2]. In our case, 57.9% had some concomitant lesions. Minguell et al. has reported that a higher rate of concomitant lesions detected in revision ACLR was associated with reduced RTS at follow-up [27]. In addition, The rate of ACL re-rupture after a revision surgery is higher than the re-rupture rate after primary reconstruction [5]. Shelbourne et al. found a reinjury rate in the first 5 years after revision surgery that ranged from 2 to 5% [28]. Our case series did not have any re-ruptures at the 2-year follow-up.

## Conclusions

In conclusion, the rates of RTS at the preinjury level following revision ACLR using autologous BPTB and modified Lemaire LET are lower than those observed after primary ACLR. Nevertheless, it is important to note that the majority, if not all, of the patients can still expect to RTS at some level. Furthermore, patients can expect significant improvements in their patient-reported functional scales, including the IKDC, Lysholm, and SF-12; with a low complications rate.

## Supplementary Information

Below is the link to the electronic supplementary material.

Supplementary file1 (XLSX 11 kb)
